# Immune factors and viral interactions in brain cancer etiology and outcomes, The 2016 Brain Tumor Epidemiology Consortium Meeting report 

**DOI:** 10.5414/NP300985

**Published:** 2016-08-22

**Authors:** Kimberly J. Johnson, Johannes A. Hainfellner, Ching C. Lau, Michael E. Scheurer, Adelheid Woehrer, Joseph Wiemels

**Affiliations:** 1 Brown School Master of Public Health Program, Washington University in St. Louis, St. Louis, MO, USA,; 2 Institute of Neurology, Medical University of Vienna, Vienna, Austria,; 3 Department of Pediatrics, Section of Hematology-Oncology, Baylor College of Medicine, Houston, TX, and; 4 Department of Epidemiology and Biostatistics, University of California, San Francisco, CA, USA

**Keywords:** brain tumors, epidemiology, immune system, cancer

## Abstract

The Brain Tumor Epidemiology Consortium (BTEC) is an international consortium that aims to advance development of multicenter and interdisciplinary collaborations that focus on research related to the etiology, outcomes, and prevention of brain tumors. The 17th annual BTEC meeting was held in Barcelona, Spain on June 21 – 23, 2016. The meeting focused on immune and viral factors that influence brain tumor development. Fundamentals of innate and adaptive immunity were reviewed, the role of immune checkpoint inhibitors in primary and secondary brain tumors was addressed, vaccination strategies for glioma treatment were presented, and the potential contribution of immune dysfunction and viruses tropic for glial cells in gliomagenesis was discussed. Further contributions addressed the risk of non-ionizing radiation, molecular and birth characteristics on brain tumor induction/outcomes, and patterns of care and effects of different treatments on brain tumor survival in the real world setting. The next BTEC meeting will be held in June 2017 in Banff, Canada, and will focus on brain tumor epidemiology in the era of precision medicine.

## Introduction 

The BTEC is an open scientific forum that fosters the development of collaborations between brain tumor researchers that will lead to a better understanding of the etiology, outcomes, and prevention of brain tumors. To attain its mission, BTEC mentors junior investigators as well as those who are new to brain tumor epidemiologic research [1]. Founded in 2003 after an initial meeting sponsored by the US National Cancer Institute’s (NCI) Division of Cancer Epidemiology and Genetics (DCEG) and the US National Institutes of Health’s (NIH) Office of Rare Diseases (ORD), the BTEC has evolved to become a self-directed consortium with working groups focused on epidemiological evaluation of adult glioma, meningioma, pediatric brain tumors, and on family-based studies of genetic susceptibility. The BTEC is a US National Cancer Institute designated consortium and a non-profit 501(c) [3] corporation. 

**Figure 1. Figure1:**
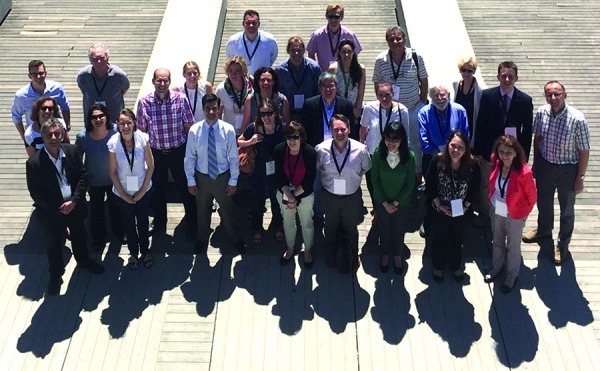
The BTEC group on its site visit of the Parc de Recerca Biomèdica de Barcelona.

The BTEC held its 2016 annual meeting in Barcelona, Spain, with the major theme “*Immune factors and viral interactions in brain cancer etiology and outcomes*”*.* In recent years the understanding of the genetics and genomics of glioma have dominated the epidemiology of glioma. Equally astounding, however, are advances in immunotherapies and yet epidemiologists have used little of this new information on glioma’s interaction with the immune system in investigations on causality. This program sought to stimulate such research. The program committee included Myrna Rosenfeld of the Institut d’Investigacions Biomèdiques August Pi I Sunyer Neuroimmunology program and Maria Martinez-Garcia of the Servicio de Oncologia Médica at the Hospital del Mar in Barcelona, Spain, along with the Board of Director members: Co-Presidents Joseph Wiemels, PhD and Adelheid Woehrer, MD, PhD; Co-Vice Presidents Johannes A. Hainfellner, MD, and Ching Lau, MD, PhD; Secretary Kim Johnson, MPH, PhD, and Treasurer Michael Scheurer, PhD, MPH. The meeting was coordinated by Ms. Bénédicte Clement of Montpellier, France. The meeting included four keynote addresses and one educational lecture with research relevant to the meeting theme and 13 abstract presentations from junior and senior brain tumor researchers. A summary of the scientific content of the meeting is provided in this report. [Fig Figure1]

## Summary of educational and keynote lectures 

**Joseph Wiemels, PhD,** of the University of California San Francisco introduced the meeting with a primer on immunology for epidemiologists and in particular research topics pertaining to brain tumors. Topics included the fundamentals of innate and adaptive immunity, antigen recognition, and glioma immunoepidemiology. Innate immunity is an evolutionarily ancient branch that depends on nonspecific patterns of invading organisms for recognition and elimination. Natural killer cells are able to recognize cancer cells from aberrant patterns of cell surface receptors and loss of major histocompatibility (MHC) antigens, an important component of anticancer activities. Adaptive immunity is facilitated by a sophisticated process of antigen receptor (immunoglobulin and T-cell receptor) creation and editing, and the orchestration of cell-to-cell communication. A key aspect of this communication is “costimulation” that refers to a second interaction between cells besides antigen recognition. Costimulation can either activate or repress an effector immune cell; this interaction is commonly referred to as a “checkpoint” and manipulation of this interaction is a strategy employed by several modern therapeutic approaches. Cancers may manipulate several branches of the immune system to create a contexture that prevents immune recognition (loss of antigens), suppresses costimulation (expression of repressive receptors), and production of immunosuppressive cytokines that promote tolerance to the tumor [2]. Epidemiologists have observed more gliomas among persons who do not have allergies and a history of weak responses to a neurotropic virus Varicella (i.e., lack of chickenpox and shingles) [3], which may be related to poor immune recognition of CNS-related antigens. Additional epidemiologic analyses relating to immune function include multiplex screening for cytokines in glioma cases and controls [4] and the enumeration of blood cell types using DNA methylation profiling [5]. 

**Matthias Preusser, MD,** spoke about “*Immune checkpoint inhibitors in primary and secondary brain tumors*.” It has long been observed that solid tumors are not only composed of cancer cells, but are generally infiltrated by immune cells. Successful manipulation of such immune cells to fight the tumor is a long-term goal of cancer immunologists. The composition and activation state of immune infiltrates is now recognized to have profound implications on patient prognosis; for instance, activated cytotoxic T-cells can fight tumors, but repressive T-regulatory cells and cytokines promote tumor tolerance and are associated with poor prognosis. The immune system is incredibly powerful, necessitating a sophisticated system of “checks and balances” for immune function control; one of these mechanisms involves balancing T-cell priming with activating and repressive receptors. Drugs were recently developed that can dampen repressive receptor interactions between cells of the immune system: these are termed checkpoint inhibitors and can act to enhance both T-cell and microglial activation against brain tumors [6]. While brain tissue is traditionally thought to be “immune privileged”, there is extensive trafficking of cells in and out as well as a lymphatic system capable of exposing professional antigen-presenting cells in lymph nodes to glioma antigens. The most promising checkpoint inhibitors target the PD-1/PD-L1 interaction, which enhances glioma therapy in both animal models and ongoing trials of newly diagnosed gliomas as well as metastatic tumors to the brain from several other cancer sites [6, 7]. Key features that correlate with efficacy include expression of PD-L1 by tumors and their antigenic load, which is reflected by a greater number of mutations in protein-coding regions. Immunotherapies are an extremely promising and a rapidly evolving aspect of brain tumor treatment. 

**Michael Weller, MD,** spoke on “*Vaccination strategies for glioma.*” Dr. Weller reviewed approaches for delivery of therapeutic vaccines, in which specific protein targets in glioma are presented to the immune system with stimulatory adjuvants in a manner to target tumor rejection. Antigens can include peptides to tumor-associated antigens (TAA) such as mutant proteins including IDH1 or overexpressed proteins such as the heat shock proteins. One of the first glioma-specific antigens identified is the EGFR variant EGFRvIII that was the subject of several early successful immunotherapy attempts [8]. This EGFR variant is expressed in ~ 50% of all EGFR-amplified tumors, leads to constitutive signaling, and itself does not have prognostic impact in patients treated with standard therapy [9]. Despite early promise in preliminary trials, an important two-arm, double-blind randomized phase III trial of the EGFRvIII vaccine (Act IV trial, CDX-110) developed in conjunction with Celldex Therapeutics has unfortunately proved to have no survival benefit. The reasons for this lack of efficacy are unclear, but correlative studies show that EGFRvIII-positive cells are dispersed throughout the tumor but appear to be lost upon disease recurrence, whereas EGFR-amplified cells (without the vIII mutation) are stable at diagnosis and relapse [9]. Despite the EGFRvIII immunotherapy failure, vaccination for glioma TAA remains a strongly viable concept and current trials are under way for several other targets. 

**Charles Cobbs, MD,** spoke on “*Evidence for CMV expression and tumor promotion in GBM: Is CMV the chicken and/or the egg?*” Dr. Cobbs outlined some of the unsettling questions about glioma, including the localized and systemic immune dysfunctions present in glioma patients. An infectious agent, especially one tropic for glial cells that induces immunosuppression mechanisms, could potentially contribute to this dysfunction. A candidate virus for these effects is cytomegalovirus (CMV) that was subsequently found present universally among gliomas [10], albeit at low levels and not by all investigators. Key characteristics of CMV-related activities mirror the “abortive lytic infection” oncogenic process that is induced by Epstein-Barr virus in lymphomagenesis, including (i) widespread infection, (ii) dysregulation of T-cell function, (iii) production of transforming cofactors such as US28, a chemokine receptor, (iv) a number of gene products that manipulate glioma biological pathways such as increased mitosis, maintenance of stemness and activated cancer signaling pathways such as AKT and RAS pathways, and (v) abortive life cycle with apparent tumor-related evolution of the virus. Investigations of gene products of CMV and animal models point to a role, and approaches to therapy using vaccines to CMV proteins show definite promise, as well as the antiviral medication valganciclovir [11]. A lively discussion followed Dr. Cobbs’ presentation in which the unanswered questions about CMV’s role in pathology and the use of new treatment modalities were covered. Conference participants agreed that continued investigations on CMV biology in glioma are highly worthwhile; however, well-designed randomized trials for CMV-based therapies are necessary particularly for antiviral therapy. 

**Elisabeth Cardis, PhD,** spoke on the topic, “*Radiation and brain tumors, current findings and research in progress.*” Dr. Cardis detailed the biological effects of radiation and the current strong evidence from studies on atomic bomb survivors, therapeutic (ringworm, cancer treatments) and diagnostic (CT scan) exposures. Evidence for non-ionizing radiation risk appears to be limited to potential tumor promotion effects induced by recent exposures only [12]. Recent interest has focused on mobile phone use, which has approached saturation in most parts of the world. While the World Health Organization now classifies mobile phone radio frequency exposures as a possible carcinogen, consistent associations between exposure and risk in large population studies is lacking and animal models have produced weak supportive mechanistic evidence. Dr. Cardis reviewed the history of ecological, cohort, and case-control studies on the subject. Ecologic studies indicate no increased risk but are only capable of detecting overall trends. Cohort studies are ongoing and require long follow-up times but so far do not indicate a strong effect. Case-control studies may suffer from selection and information bias, but do suggest that ionizing radiation increases risk to brain tumors at high RF exposure levels [13]. Validation studies are concentrating on additional factors, such as laterality of the tumor, anatomical location, and more precise dose estimations; these studies provide some consistent results indicating a possible risk associated with the use of mobile phone. As mobile phone use continues to increase and evolve, continuing studies that determine exposure, risk assessments, are essential – several studies on children (Mobi-Kids) [12], novel methodologies (GERoNiMO), and occupational exposures (INTEROCC) [14] are poised to contribute additional findings in short order. 

**Abstract Presentations.** There were 13 abstracts presented over two days that spanned basic to population sciences and included talks covering mechanisms of disease progression, molecular and birth characteristics on brain tumor risk/outcomes, and descriptive epidemiology of patterns of care and the effects of different treatments on survival. 

The abstract session opened with two junior investigator award presentations that were sponsored by the American Brain Tumor Association. The first presentation was by **Magdalena Neuhauser** of the Institute of Neurology at the Medical University of Vienna, Austria. In her talk titled: “*ABTR-SANO Real-World Pattern of Care Study on Primary Central Nervous System Lymphoma*”, she described the results of a study of patterns of cancer (POC) in 77 patients with primary central nervous system lymphoma (PCNSL) that were treated at several Austrian institutions. Ms. Neuhauser reported highly variable treatment protocols across institutions. She also noted from her study that patients treated with first-line chemotherapy or combined therapy had better outcomes than those treated with radiotherapy. She concluded that her study could serve as a reference for future assessments of the implementation of treatment guidelines into practice. The second junior investigator award presentation was given by **Steven Francis, PhD,** of the University of California San Francisco. In his talk titled “*Germline Variation of Human Endogenous Retroviruses: An Etiologic role in Glioblastoma?*”, he described results from a foundational study of whole genome sequencing data from 2,557 individuals from the 1,000 Genomes Phase 3 project and from 52 individuals with glioblastoma from The Cancer Genome Atlas (TCGA). Dr. Francis described patterns of human endogenous retrovirus K (HERV-K the most recent viral insertion in humans) insertion. Using a custom bioinformatic pipeline, Dr. Francis reported identifying 1,788 unique HERV-K insertion sites across 2,557 genomes, including 251 that were not represented in the reference genome hg19. Dr. Francis also reported that there is significant heterogeneity in HERV-K insertions both within and between human populations and that the prevalence of insertion seems to vary between the 1,000 genomes cohort and the TCGA cohort with glioblastoma. He concluded that further study is needed to determine if the HERV-K insertions are an etiologic factor for glioblastoma. 

**Jill Barnholtz-Sloan, PhD,** of Case Western Reserve University, School of Medicine gave a talk titled “*Role of Neutrophil-to-Lymphocyte Ratio and Platelet-to-Lymphocyte Ratio in Malignant Behavior and Prognosis in Lower and High Grade Glioma.*” Dr. Barnholtz-Sloan presented results from a study that assessed whether neutrophil-to-lymphocyte ratio (NLR) and platelet-to-lymphocyte ratio (PLR) were associated with clinical outcomes in 206 Ohio Brain Tumor Study patients. The major finding from her study was that NLR and PLR are not significant predictors of overall survival or time to recurrence in Grade II – IV glioma patients, but further research with larger sample sizes is warranted. The next talk titled “*IDH mutation and 1p/19q codeletion distinguish two radiological patterns of diffuse low-grade gliomas*” was given by **Amelie Darlix, MD,** of the Montpellier Cancer Institute in France. Dr. Darlix described findings from a study of 198 patients with diffuse low-grade glioma (DLGG) that aimed to determine whether IDH and 1p/19q codeletions were correlated with radiological characteristics of the tumor. Dr. Darlix’s study showed that IDH wild-type, 1p/19q non-codeleted tumors are radiologically distinct from those that are IDH-mutated and 1p/19q codeleted. 

**Abderrezak Giodouche, PhD,** of the University of Bejaia, Algeria, gave the last talk of the first day titled “*Retrospective epidemiological study of brain tumors in Algeria: Case of Bejaia State.*” Dr. Ghidouche described the results of an epidemiological study of primary and secondary brain tumors in the neurosurgery department of Bejaia University Hospital from 2012 to 2015. The most interesting finding was that the median age at diagnosis (46 years) is much lower than in the rest of the world (58 years) but is consistent with findings from other North African countries. 

**Andreas Hainfellner **presented the first abstract talk on the second day titled “*ABTR-SANO Real-World Pattern of Care Study on Glioblastoma in the Austrian Population.*” Mr. Hainfellner presented POC results for GBM cases seen at all Austrian neuro-oncology units. He provided background information that state-of-the-art treatment for glioblastoma consists of maximal safe resection followed by concurrent use of temozolomide during radiation. His study indicated that the current standard of care has been widely adopted across Austrian neuro-oncology units. **Jordan Ross** next presented a talk on “*Urban-Rural Residence and Brain Cancer Survival in Canada: 1996 – 2008.*” The objective of the study was to examine whether urban-rural residence affects brain cancer survival in Canada. The study used population-based data from the Canadian Cancer Registry on patients diagnosed with primary brain tumors from 1996 to 2008.The main study finding was no significant difference in survival according to urban-rural residence. **Jordan Ross’** second talk was titled “*Conditional Survival after Diagnosis with Malignant Brain and Central Nervous System Tumours in Canada: 2000 – 2008.*” The goal of this study was to provide the first population-based estimates of conditional survival rates for brain and central nervous system tumors in Canada. Conditional survival rates are defined as the probability that a person will survive an additional amount of time given that they have survived a defined period post-diagnosis, which can be useful for predicting individual patient prognosis. The study included 20,875 patients with brain tumor diagnoses from 2000 to 2008. The main findings from the study were an improvement in survival with increased time since diagnosis for nearly all histology subtypes. The fourth talk of this session was given by **Kimberly Johnson, MPH, PhD,** of Washington University on “*Peri-gestational risk factors for pediatric brain tumors in Neurofibromatosis Type 1.*” Dr. Johnson reported the results from a cross-sectional study of 606 individuals < 18 years old who were enrolled in the Neurofibromatosis Type 1 (NF1) Patient Registry Initiative from 6/9/2011 to 6/29/2015. Dr. Johnson reported a significantly higher birth weight in individuals with optic pathway glioma (OPG) diagnoses than in those without OPG, which is consistent with published results from studies of pediatric brain tumors in the general population. These results suggest that factors influencing prenatal growth may also influence tumor development in children with NF1 [15]. **Todd Druley, MD, PhD,** also of Washington University in St. Louis next presented a talk titled “*Childhood malignancies are more prevalent in males than females with congenital anomalies.*” Using retrospective clinical data from a large academic research center, 479 pediatric malignancy diagnoses were identified with an approximately equal distribution of males and females. A total of 85 of these children had a congenital anomaly. CNS anomalies were the most prevalent anomalies with significantly more males having CNS anomalies than females and the majority of CNS anomaly cases having CNS tumors. Dr. Druley concluded that these data provide a foundation for further research aimed at understanding the mechanism for the sex difference. **Joseph Wiemels, PhD,** of UCSF gave a talk on “*Protein-specific antibody responses to Varicella and Cytomegalovirus in glioma patients compared to controls.*” In Dr. Wiemels study, antibody presence against 99 varicella (VZV) and 45 cytomegalovirus (CMV) proteins were examined using Nucleic Acid Programmable Protein Arrays (NAPPA) in serum samples from 45 glioma patients and 45 controls. Cases were more likely to have complex reactions to ≥ 5 VZV antigens. For CMV, cases also reacted stronger to specific CMV proteins. Further research is planned to confirm these results in prediagnostic sera from glioma cases and matched controls as well as studies to determine if antibody reactions affect glioma survival. **Michael Scheurer, PhD** of Baylor College of Medicine presented a study on behalf of Austin Brown also of Baylor, titled “*Epigenome-wide association study identifies novel susceptibility loci for treatment-related ototoxicity among survivors of pediatric medulloblastoma.*” The study included data from a discovery and replication cohort of 62 and 18 medulloblastoma survivors respectively who were treated with cisplatin chemotherapy regimens from 2005 to 2012. The investigators used Illumina HumanMethylation450 BeadChip arrays to examine methylation patterns. The authors identified a novel CpG methylation locus associated with ototoxicity severity that corresponded to increased gene expression of *PAK4*, a regulator of apoptosis that has also been implicated in cisplatin resistance in malignant cell lines. Further research is needed to establish a mechanism. The final talk of the abstract session titled “*A nested case-control study of pre-diagnostic serum cytokines and glioma*“ was given by **Judith Schwartzbaum, PhD,** of The Ohio State University. The goal of this study was to determine if 277 pre-diagnostic serum cytokines were associated with glioma risk and whether the tumor influences correlations among serum cytokines near the time of diagnosis. Serum samples from 487 glioma patients and 487 matched controls that were obtained from the Janus Serum bank were included. The investigators used logistic regression to determine differences in prediagnostic cytokines between cases and controls and to compare cytokine correlation patterns for 12 cytokines between cases and controls for varying periods before case diagnosis. sIL10RB, CCL22, β-catenin, IL4*sIL4RA were associated with glioma for > 20 years prior to diagnosis. Cytokine correlations within 5 years of diagnosis were weaker in cases than controls for the 12 cytokines as well as all 277 cytokines. Dr. Schwartzbaum concluded that serum cytokines affect glioma risk and that the pre-diagnostic tumor may induce immune suppression within 5 years prior to glioma diagnosis. 

## Conclusions 

The meeting was attended by scientists with diverse disciplinary backgrounds. Brain tumor development and prognosis are influenced by a multitude of factors, emphasizing the need to integrate different viewpoints into epidemiological discussions of potential causes and factors impacting prognosis. A role for immune factors is clearly evident for glioma pathophysiology, and is likely also important for etiology. At least two viral infections are under investigation for their role in gliomagenesis, and epidemiologists will be wise to pursue further research incorporating measures of immune function into their research projects. The 2016 meeting underscored the multi-disciplinary nature of BTEC. BTEC invites all investigators with an interest in identifying brain tumor causes and factors affecting survival to join BTEC and to attend future meetings. 

## Acknowledgments 

We are thankful for the generous meeting support by the Parc de Recerca Biomèdica de Barcelona and the American Brain Tumor Association. Funding for this conference was also made possible (in part) by 1R13CA206359-01 from the National Cancer Institute to Joseph Wiemels, PhD. The views expressed in written conference materials or publications and by speakers and moderators do not necessarily reflect the official policies of the Department of Health and Human Services; nor does mention by trade names, commercial practices, organizations imply endorsement by the US Government. 

## Conflict of interest 

All authors declare that there are no conflicts of interest. 

## References

[1] Johnson KJ Scheurer ME Woehrer A Wiemels J Evolving evidence on tumor and germline genetic classification of gliomas: implications for etiology and survival studies. Clin Neuropathol. 2016; 35: 31–38. 26588027 10.5414/NP300917

[2] Fridman WH Pagès F Sautès-Fridman C Galon J The immune contexture in human tumours: impact on clinical outcome. Nat Rev Cancer. 2012; 12: 298–306. 22419253 10.1038/nrc3245

[3] Amirian ES Scheurer ME Zhou R Wrensch MR Armstrong GN Lachance D Olson SH Lau CC Claus EB Barnholtz-Sloan JS Il’yasova D Schildkraut J Ali-Osman F Sadetzki S Jenkins RB Bernstein JL Merrell RT Davis FG Lai R Shete S History of chickenpox in glioma risk: a report from the glioma international case-control study (GICC). Cancer Med. 2016; 5: 1352–1358. 26972449 10.1002/cam4.682 PMC4924393

[4] Schwartzbaum J Seweryn M Holloman C Harris R Handelman SK Rempala GA Huang RP Burkholder B Brandemihl A Kallberg H Johannesen TB Ahlbom A Feychting M Grimsrud TK Association between Prediagnostic Allergy-Related Serum Cytokines and Glioma. PLoS One. 2015; 10: e0137503. 26352148 10.1371/journal.pone.0137503 PMC4564184

[5] Koestler DC Jones MJ Usset J Christensen BC Butler RA Kobor MS Wiencke JK Kelsey KT Improving cell mixture deconvolution by identifying optimal DNA methylation libraries (IDOL). BMC Bioinformatics. 2016; 17: 120. 26956433 10.1186/s12859-016-0943-7 PMC4782368

[6] Preusser M Lim M Hafler DA Reardon DA Sampson JH Prospects of immune checkpoint modulators in the treatment of glioblastoma. Nat Rev Neurol. 2015; 11: 504–514. 26260659 10.1038/nrneurol.2015.139 PMC4782584

[7] Berghoff AS Venur VA Preusser M Ahluwalia MS Immune Checkpoint Inhibitors in Brain Metastases: From Biology to Treatment. Am Soc Clin Oncol Educ Book. 2016; 35: e116–e122. 27249713 10.1200/EDBK_100005

[8] Choi BD Archer GE Mitchell DA Heimberger AB McLendon RE Bigner DD Sampson JH EGFRvIII-targeted vaccination therapy of malignant glioma. Brain Pathol. 2009; 19: 713–723. 19744042 10.1111/j.1750-3639.2009.00318.x PMC2846812

[9] Weller M Kaulich K Hentschel B Felsberg J Gramatzki D Pietsch T Simon M Westphal M Schackert G Tonn JC von Deimling A Davis T Weiss WA Loeffler M Reifenberger G Assessment and prognostic significance of the epidermal growth factor receptor vIII mutation in glioblastoma patients treated with concurrent and adjuvant temozolomide radiochemotherapy. Int J Cancer. 2014; 134: 2437–2447. 24614983 10.1002/ijc.28576

[10] Cobbs CS Harkins L Samanta M Gillespie GY Bharara S King PH Nabors LB Cobbs CG Britt WJ Human cytomegalovirus infection and expression in human malignant glioma. Cancer Res. 2002; 62: 3347–3350. 12067971

[11] Cobbs CS Cytomegalovirus and brain tumor: epidemiology, biology and therapeutic aspects. Curr Opin Oncol. 2013; 25: 682–688. 24097102 10.1097/CCO.0000000000000005

[12] Turner MC Benke G Bowman JD Figuerola J Fleming S Hours M Kincl L Krewski D McLean D Parent ME Richardson L Sadetzki S Schlaefer K Schlehofer B Schüz J Siemiatycki J van Tongeren M Cardis E Occupational exposure to extremely low-frequency magnetic fields and brain tumor risks in the INTEROCC study. Cancer Epidemiol Biomarkers Prev. 2014; 23: 1863–1872. 24935666 10.1158/1055-9965.EPI-14-0102 PMC4154968

[13] Group IS Brain tumour risk in relation to mobile telephone use: results of the INTERPHONE international case-control study. Int J Epidemiol. 2010; 39: 675–694. 20483835 10.1093/ije/dyq079

[14] Vila J Bowman JD Richardson L Kincl L Conover DL McLean D Mann S Vecchia P van Tongeren M Cardis E A Source-based Measurement Database for Occupational Exposure Assessment of Electromagnetic Fields in the INTEROCC Study: A Literature Review Approach. Ann Occup Hyg. 2016; 60: 184–204. 26493616 10.1093/annhyg/mev076 PMC4738235

[15] Johnson KJ Zoellner NL Gutmann DH Peri-gestational risk factors for pediatric brain tumors in Neurofibromatosis Type 1. Cancer Epidemiol. 2016; 42: 53–59. 27018750 10.1016/j.canep.2016.03.005 PMC4899111

